# Peptides encoded by noncoding genes: challenges and perspectives

**DOI:** 10.1038/s41392-019-0092-3

**Published:** 2019-12-13

**Authors:** Shuo Wang, Chuanbin Mao, Shanrong Liu

**Affiliations:** 1 Changhai Hospital, Shanghai, 200433 China; 2 Department of Chemistry and Biochemistry, Stephenson Life Sciences Research Center, Institute for Biomedical Engineering, Science and Technology, University of Oklahoma, 101 Stephenson Parkway, Norman, OK 73019-5300 USA

**Keywords:** Genome, Drug discovery, Biochemistry, Cancer, Therapeutics

## Abstract

In recent years, noncoding gene (NCG) translation events have been frequently discovered. The resultant peptides, as novel findings in the life sciences, perform unexpected functions of increasingly recognized importance in many fundamental biological and pathological processes. The emergence of these novel peptides, in turn, has advanced the field of genomics while indispensably aiding living organisms. The peptides from NCGs serve as important links between extracellular stimuli and intracellular adjustment mechanisms. These peptides are also important entry points for further exploration of the mysteries of life that may trigger a new round of revolutionary biotechnological discoveries. Insights into NCG-derived peptides will assist in understanding the secrets of life and the causes of diseases, and will also open up new paths to the treatment of diseases such as cancer. Here, a critical review is presented on the action modes and biological functions of the peptides encoded by NCGs. The challenges and future trends in searching for and studying NCG peptides are also critically discussed.

## Introduction

The central dogma of molecular biology describes the basic principles of the transfer of genetic information between biological macromolecules in cells. Genetic information flows from genes to proteins, which comprise the material basis of life and are the main participants in life activities.^1,2^ Protein-coding genes make up <3% of the human genome, and only a small fraction in the remaining 97% of the genome (composed of noncoding genes, NCGs) is characterized.^3^ Many NCGs were previously defined as junk DNA, but they are truly functional elements.^4^ The emergent discovery of noncoding RNA returned NCGs into the focus of life scientists, encouraging them to view NCGs from a new perspective. Noncoding RNA plays a broad and important role in regulating gene expression and various life activities through the formation of RNA–protein complexes^5,6^ or through base complementation.^7,8^ Noncoding RNA is classified into many categories. Small nuclear RNA has been a recognized noncoding RNA for a relatively long time. Its main function is to participate in the processing of mRNA precursors. The RNA components in splicing bodies such as U1, U2, U4, and U6 are small nuclear RNAs.^9^ MiRNAs constitute a class of single-stranded RNA molecules encoded by endogenous genes, and are ~22 nucleotides in length. They are involved in the regulation of posttranscriptional gene expression. They can bind to the untranslated region (UTR) of target gene mRNA from which it guides either an RNA-induced silencing complex (RISC) to prevent mRNA translation or AGO proteins to cleave mRNA, to achieve endogenous gene expression.^10,11^ CircRNA was first discovered in viroids, in which the genome is a single-stranded circular RNA molecule.^12^ CircRNAs can act as molecular sponges to counteract the role of miRNAs. CircRNAs can also act as scaffolds for different molecular interactions.^13^ Long noncoding RNAs (lncRNAs) are considered noncoding because they lack obvious long protein-coding open-reading frames (ORFs), although new evidence shows that some lncRNAs are truly coded into proteins. LncRNAs have been proposed to have diverse functions, including transcriptional regulation, organization of nuclear domains, and regulation of gene expression.^14^ Currently, the NCG revolution has been leveraged to study all living organisms.^15,16^

**Fig. 1:**
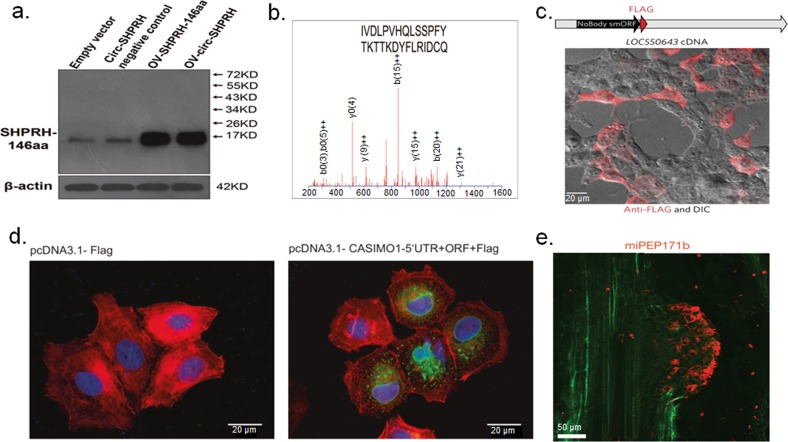
Many laboratory techniques support the idea that peptides derived from noncoding genes exist. **a** Design of antibodies against NCG peptides and verification by western blot analysis.^38^ **b** Mass spectrometry is used to identify specific signals of noncoding gene peptides.^39^ **c** Immunofluorescence images of peptide-FLAG fusion protein (red, NCG-peptide NoBody; DIC, differential interference contrast).^75^ **d** Immunofluorescence images showing expression of the FLAG-tagged NCG peptide (green, NCG-peptide CASIMO1; red, actin filaments; blue, nucleus).^98^ **e** Images of the immunolocalization of the NCG peptides (red, NCG-peptide miPEP171b; green, autofluorescence).^113^

Moreover, with the development of technologies such as ribosome profiling and high-throughput sequencing in addition to protein database searches for large-scale proteomic analysis, some novel peptide annotations have been found that do not match currently annotated protein-coding genes; in contrast, they correspond to the genes of noncoding RNAs, pseudogenes, UTRs, etc., which were previously considered to be NCGs.^17,18^ Recently, an increasing number of experiments have indicated that NCGs can indeed be translated,^19,20^ and that the translation products are mainly polypeptides or micropeptides.^21,22^ NCG peptides can be directly verified by western blotting (WB) using specific antibodies. In addition, NCG peptides can be combined with epitope tags such as FLAG, human influenza hemagglutinin (HA), or green fluorescent protein (GFP) to form fusion proteins. The resultant fusion proteins can be detected through WB or fluorescence imaging technology. Mass spectrometry techniques, such as liquid chromatography with a tandem mass spectrometer, can also confirm the presence of NCG peptides by analyzing the signals of the NCG peptides (Fig. 1). These peptides have a wide range of biological functions. Interestingly, some NCG peptides have significant tissue-specific distribution patterns and can undertake finely tuned local regulation in a tissue-specific manner.

**Fig. 2:**
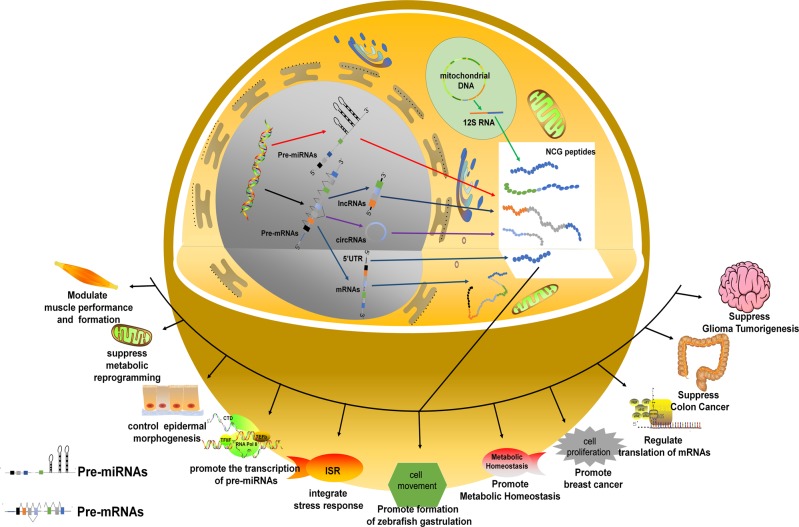
The biological functions of NCG peptides. The expression of noncoding genes is achieved through central rules. After transcription, alternative splicing results in a variety of transcripts, some of which are translated into peptides. These peptides play important roles in modulating muscle formation and performance, suppressing metabolic reprogramming, controlling epidermal morphogenesis, promoting pre-miRNA transcription, regulating mRNA translation, integrating aspects of the stress response, facilitating gastrulation formation, enhancing metabolic homeostasis, and inducing or suppressing tumorigenesis and/or tumor progression.

In this review, we summarize the structure, action modes, and biological roles of peptides derived from NCGs (Fig. 2). The NCG-derived peptides (termed NCG peptides) discovered thus far are summarized in Table 1, and are critically discussed in this review. The appearance of these peptides suggests that a portion of the genome that encodes proteins or peptides is much larger than that previously recognized. Finally, we address the biological and medical significance of NCG peptides and propose future directions for studying NCG peptides to advance the field. We believe that a deeper exploration into this subject will explain some mysteries of life more precisely and in greater detail, and thus lead to new biomarkers for disease diagnosis and therapeutics.

**Table 1:** NCG peptides summary

NCG-peptide name	Corresponding NCG or transcript	Functions	References
SHPRH-146aa	Circ-SHPRH	Down-regulation of the ubiquitination of SHPRH, and suppression of glioma tumorigenesis	^38^
FBXW7-185aa	circRNA-FBXW7	Down-regulation of the ubiquitination of FBXW7α, and suppression of glioma tumorigenesis	^39^
β-catenin-370aa	circβ-catenin	Protecting full-length β-catenin from being phosphorylated and consequently degraded, and promoting liver cancer growth and metastasis	^40^
Myoregulin (MLN)	LINC00948	Modulation of the intracellular calcium handling and muscle performance	^41^
Fungal arginine attenuator peptide (AAP)	5′ uORF in the mRNA of CPS-A small subunit	Stalling translation	^42^
pri peptide	Drosophila's MRE29 gene	Temporal control over epidermal morphogenesis	^46^
MOTS-c	Mitochondrial 12 S rRNA	Cellular and organismal metabolic homeostasis	^51^
Toddler peptide	Toddler RNA	Activation of APJ/Apelin signaling and promotion of cell movement during zebrafish gastrulation	^52^
Minion/myomixer	transcript of Gm7325	Promotion of the fusion of mononuclear myoblasts	^65,66^
MPM/Mtln/MOXI	1500011K16Rik/ LINC00116	Enhancing mitochondrial respiratory activity and fatty acid β-Oxidation, as well as promoting myogenic differentiation.	^69,77,78^
DWORF	LOC100507537	Enhancement of SERCA activity in muscle	^74^
NoBody	LINC01420/LOC550643	Inhibition of nonsense-mediated decay	^75^
Nameless peptide 1	uORF in the 5'UTR of mRNA of CHOP	Inhibition of the translation of CHOP protein in calm	^83^
Nameless peptide 2	uORF in IFRD1 mRNA	Cause of IFRD1 mRNA instability in resting cells	^86^
Nameless peptide 3	uORF in mRNA of BiP	Assistance of the translation of Bip during stress	^29^
SPAR peptide	LINC00961	Suppression of mTORC1 activation in response to amino acid stimulation	^89^
HOXB-AS3 peptide	lncRNA HOXB-AS3	Inhibition of the formation of PKM2 and suppressing tumor formation	^96^
circPPP1R12A-73aa	circPPP1R12A	Activating the Hippo-YAP signaling pathway and enhancing the tumorigenesis and metastasis of colon cancer	^97^
CASIMO1 peptide	noncoding RNA (NR_029453)	Promotion of the cell proliferation and migration in breast cancer	^98^
PINT87aa	circPINTexon2	Inhibiting several oncogenes and suppressing glioblastoma	^106^
miPEP165a	pri-miR165a	Promotion of the transcription of pre-miR165a	^115^
miPEP171b	pri-miR171b	Promotion of the transcription of pre-miR171b	^115^
STORM	linc00689	Competition for *7SL* RNA	^126^

### Action modes of NCG-derived peptides

#### NCG peptides are different from traditional proteins in hierarchical structures

The correct spatial folding of protein structures is the basis of formal biological function.^23^ The spatial conformation of the protein is described with four hierarchical structures. The primary structure, i.e., the order of the amino acid residues from the N-terminus to the C-terminus, is determined by the order of nucleic acid in the corresponding genes. On the basis of the primary structure, atoms on the peptide chain backbone form local substructures, known as the secondary structure. Several consecutive secondary structures can be combined into a “supersecondary unit”, and a plurality of such units further form a “structural domain”, which constitutes the tertiary structure.^24,25^ The structural domain is self-stabilizing and prominent such that the host proteins can maintain proper biological function.^26,27^ The tertiary structure is the spatial arrangement of all the atoms in one peptide chain. In the traditional sense, a protein is determined by the formation of a tertiary structure. The spatial arrangement and functional cooperation of the subunits result in the quaternary structure.^28^ The length of most NCG peptides contains fewer than 100 amino acid residues (aa), with the shortest being only 9 aa long.^29^ The number of amino acids is the basis for the formation of complex protein structures. To form even the simplest transmembrane α-helix (TMH) structure, 30 amino acids are needed, and unstructured spacer regions between different structures in the protein are also required.^30^ Hence, in contrast to conventional proteins, NCG peptides usually do not form a complicated structure, but have different modes of action, as described below. Although some circRNA-derived NCG peptides are composed of >100 aa, they are much smaller than most traditional proteins (for example, FBXW7 has 185 aa and β-catenin has 370 aa). Considering that most circRNAs are derived from exons, more evidence is needed to determine whether some circRNAs can be classified as other types of messenger RNA. The recently discovered circRNA-derived NCG peptides with clear mechanisms of action tend to function through interactions with other proteins and their mechanisms that are also discussed below.

#### NCG peptides function in a sequence-independent or sequence-dependent manner

**Fig. 3:**
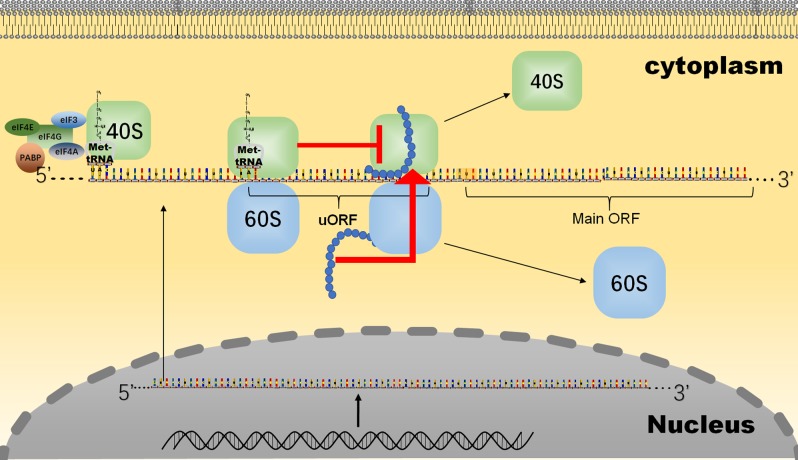
Scanning PICs that participate in the translation of uORFs can be reinitiated at the ORF in the coding region.

**Fig. 4:**
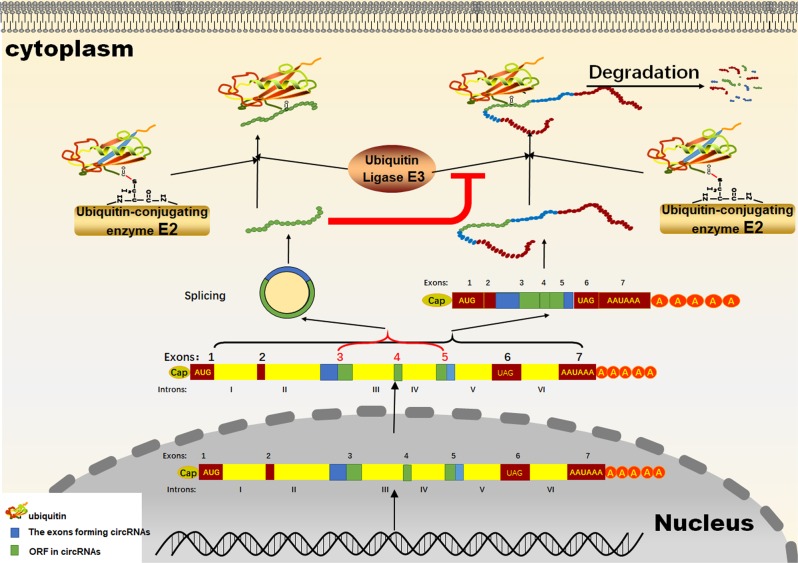
Action mode of circRNA-derived peptides. CircRNA-derived peptides downregulate the ubiquitination of the full-length protein derived from the same maternal gene as a competitive inhibitor, which results in the accumulation of full-length proteins and the consequent effects.

Scanning by the 40S–Met-tRNAi complex (43S complex) is the major process before translation initiation and involves binding to mRNA.^31,32^ A part of a polypeptide is translated from an upstream open-reading frame (uORF) in the 5′UTR and is conserved among species according to phylogenetic analysis.^33^ A class of regulatory peptides translated from uORFs creates a peptide-sequence-independent ambuscade for the 43S complex, as it seeks a downstream start codon (Fig. 3). Through this ambuscade, the scanning process is blocked. However, a sequence-dependent approach is more common. Some NCG peptides can act as competitive inhibitors through the same sequence as the proteins with which they are homologous. Many of the circRNAs are derived from the back-spliced exon of their maternal genes.^34,35^ Therefore, different RNA forms of the same gene share partially repeated sequences that encode polypeptides. For example, the SNF2 histone linker PHD RING helicase (SHPRH)-146aa (Table 1) is a peptide translated from a cirRNA. Full-length SHPRH, encoded by the maternal gene of Circ-SHPRH, is an E3 ligase. It promotes ubiquitinated proteasome-mediated degradation of proliferating cell nuclear antigen (PCNA), which leads to inhibited cell proliferation.^36,37^ Another E3 ligase, denticleless E3 ubiquitin protein ligase (DTL), induces the ubiquitination of SHPRH. Two sites (K1562 and K1572) of DTL-initiated ubiquitination in SHPRH are also found in SHPRH-146aa. Therefore, SHPRH-146aa acts as a competitive inhibitor to suppress the ubiquitination of SHPRH, which results in the accumulation of SHPRH and the subsequent degradation of PCNA.^38^ The peptide translated from the circRNA of FBXW7 was named FBXW7-185aa (Table 1). FBXW7-185aa induces the accumulation of FBXW7α and the degradation of C-myc through the same mechanism as that used by SHPRH-146aa.^39^ Circ-0004194 originates from the β-catenin gene locus and is also known as circβ-catenin. Circ-0004194 can produce a a β-catenin isoform comprising 370 aa, termed β-catenin-370aa. β-catenin-370aa serves as an effective competitor by binding GSK3β to protect full-length β-catenin from being phosphorylated and subsequently degraded (Fig. 4).^40^

#### NCG peptides function by binding other proteins to change their conformation

Myoregulin (MLN) (Table 1) is translated from LINC00948, and the small open-reading frame (sORF) encoding MLN is located on exon 3 in the parent gene of LINC00948. The secondary structure of MLN contains a C-terminal transmembrane alpha helix. The output of computational molecular modeling demonstrates that the α-helix interacts directly with the groove jointly shaped by the M2, M6, and M9 spirals in sarco-endoplasmic reticulum Ca^2+^-ATPase (SERCA) to modulate intracellular calcium metabolism.^41^ In addition to the biochemical data, cryo-electron microscopy has revealed the action mode of fungal arginine attenuator peptide (AAP) (Table 1) directly from a structural perspective. AAP is encoded by an uORF and can lead to stalled translation.^42^ Cryo-electron microscopy has shown that AAP interacts directly with ribosome tunnel components, including RNAs and proteins, which are sandwiched between residues L4 and L17 in the large subunit.^43,44^ Mutations in AAP residues that interact directly with the ribosome can abolish the stalling effect. In addition, the C-terminus of the AAP forms a helix, which may contribute to the conformational change that accommodates the peptidyl transferase center (PTC). Through the direct interaction of secondary structures, AAP changes the conformation of the PTC, causing translational stalling. NCG peptides can act as domain-specific adapters in addition to inducers of conformation changes of other proteins. The Drosophila MRE29 gene is considered a NCG and is also known as pri (polished rice).^45^ In fact, pri encodes a 11–32 aa polypeptide (Table 1).^46^ At the 13–16-day stage of embryonic development, pri peptides are expressed and act as a specific adapter that mediates the specific binding of E3 ligase Ubr3 to the N-terminus of Shavenbaby (Svb). Consequently, the N-terminus of the ubiquitinated Svb is truncated by a proteasome. In addition, two folded regions in the C-terminus prevent Svb from complete degradation.^47^ Pri peptides contribute to proper Svb processing and convert the suppressed Svb into an active form.

#### NCG peptides act as signaling pathway molecules

In humans, the mitochondrial genome is a circular and closed genetic system that includes encoding genes of 13 proteins and NCGs of rRNAs and tRNAs.^48,49^ However, previously unknown transcripts of nuclear and small RNAs were recently discovered in the mitochondria.^20,50^ Furthermore, there is a sORF in mitochondrial 12S rRNA that can be translated into a peptide of 16 aa, named MOTS-c (Table 1). MOTS-c inhibits the folate cycle, leading to accumulating AICAR (5-aminoimidazole-4-carboxamide ribonucleotide), which can activate the AMPK pathway. Through this signaling pathway, MOTS-c has an extensive impact on cellular and organismal metabolic homeostasis.^51^ Toddler RNA, also known as Apela/Elabela/Ende, which was initially considered a noncoding RNA, encodes a peptide (Table 1). Toddler peptide activates APJ/Apelin signaling by driving the internalization of G protein-coupled Apelin receptors and promotes cell movement during zebrafish gastrulation.^52^

In contrast to being the primary inducers of biological activity, these structurally simple peptides encoded by NCGs have more of a fine-tuning effect through many different mechanisms. Because of the particularities of the NCG-peptide origins, some action modes can be said to be unique, such as those of competitive inhibitors. The finely tuned regulation of these peptides enables the living body to perform various functions more accurately and stably.

### Regulation of NCG-peptide expression

Peptides derived from NCGs are also regulated at all levels from translation to protein modification. Since many NCGs are noncoding RNAs, the regulation of their transcription is not discussed. At the translational level, abundant methylation modifications in circRNAs can enhance the level of their translation activitiy. Under some conditions, the m6A marks abundant near the start codon indicate circRNA methylation. YTHDF3 recognizes the methylated modification and promotes translation in an eIF4G2-associated cap-independent manner. In addition, circRNA translation is increased under heat-shock conditions.^53^ Similar mechanisms in the regulation of mRNA translation have been discovered, providing a model for selective mRNA translation during stress.^54,55^ Poly(A) or poly(T) sequences after a stop codon can inhibit circRNA translation, suggesting that NCG peptides are different from traditional proteins at the translational level.^56^

**Fig. 5:**
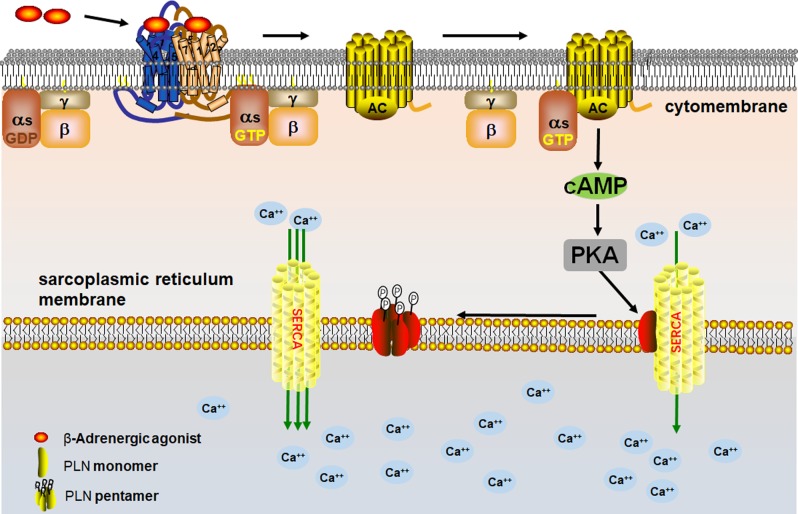
Regulation of NCG peptides. The β-adrenergic agonist phosphorylates PLN monomers to form a pentamer, thereby suppressing the inhibition of PLN on SERCA and promoting myocardial contractility.

At the level of protein modification, PLN and SLN, which have very similars to that of MLN and distinct tissue-specific distribution patterns, were originally discovered as micropeptides.^57,58^ PLN functions through the physical formation of combinations, and its function is regulated by phosphorylation and dephosphorylation in vivo. Dephosphorylated PLN mainly exists in the form of a monomer, inhibiting cardiac function by inhibiting SERCA, which is located in the sarcoplasmic reticulum (SR) membrane, and pumps Ca^2+^ from the cytoplasm back through the SR during muscle relaxation. After phosphorylation, PLN forms pentamers, which reduce the inhibitory effect on SERCA.^59^ This dynamic balance plays a key role in the enhancement of myocardial function by β-adrenergic agonists (Fig. 5). In addition, a specific PLN mutant (R9C), in which residue 9 is a mutated, inhibits phosphorylation of wild-type PLN and therefore chronically inhibits SERCA. Consequently, chronic inhibition causes dilated cardiomyopathy and premature death.^60^ In another case, that of the R14del mutant, the mutant PLN appears in the sarcolemma by mistake, where it interacts with Na/K-ATPase, resulting in cardiac remodeling, despite enhanced contractility.^61^ Orderly regulation indicates that the polypeptides derived from NCGs are inherent participants in life activities.

### Biological functions of NCG peptides

Although the number of coding genes in a eukaryotic organism is not significantly larger than that in a prokaryotic organism, the physiological and pathological activities in the eukaryotic organism are more complex than those in the prokaryotic organism. NCGs are thought to play a pivotal part in establishing this difference between eukaryotes and prokaryotes. In recent years, continuous research has demonstrated that NCG-derived peptides have considerable biological functions covering various fields. The manner in which NCG peptides establish the differences between eukaryotes and prokaryotes is discussed in greater detail below.

#### NCG peptides facilitate embryonic development

Embryonic development requires that genes are expressed in an orderly manner.^62^ This process is called genetic programming and involves multifaceted regulation.^63^ Peptides derived from NCGs can regulate this process temporally. For example, the above-mentioned pri peptide shows tissue- and time-specific expression during embryogenesis, and its knockout is lethal to embryonic development.^46^ Expressed Svb remains in a state of inhibition until pri peptide expression is initiated.^64^ Therefore, the pri peptide provides accurate temporal control over epidermal morphogenesis. Similarly, the transcript of Gm7325 in human beings is annotated as a long noncoding RNA (lncRNA), and in fact, it can be translated into an 84-amino acid polypeptide Minion,^65^ also named Myomixer (Table 1).^66^ The expression of Myomixer/Minion is upregulated during the differentiation of C2C12 myoblasts, and downregulated following myoblast fusion. In terms of a mechanism of action, Minion together with Myomaker promotes the fusion of mononuclear myoblasts, which is essential for skeletal muscle formation during embryogenesis. Although Myomixer/Minion does not affect the expression levels of the Myomaker, Myomaker cannot induce myocyte fusion in the absence of Myomixer. Combined with the time specificity of expression, Myomixer/Minion functions as a Myomaker switch that acts synergistically at a specific time point.^67,68^ Another micropeptide, MPM (micropeptide in mitochondria), is also produced by lncRNA 1500011K16Rik (in mice) or LINC00116 (in humans). MPM, also known as mitoregulin (Mtln), promotes myogenic differentiation and has an inducive effect on muscle growth and regeneration. In terms of mechanisms, the ectopic expression of genes that enhance mitochondrial respiration can rescue the phenotype induced by MPM interference, thus providing evidence that the effect of MPM in muscle tissue development and postinjury regeneration is related to the role of MPM in mitochondrial respiration.^69^ In addition, functioning as a signaling pathway molecule, Toddler peptide (also known as Apela) (Table 1) activates APJ/Apelin signaling to promote gastrulation movements,^52^ and regulates mesodermal cell migration downstream of Nodal signaling in zebrafish.^70^ Loss-of-function assays using CRISPR/Cas9 suggest that Apela also has an extenive impact on mouse embryo development.^71^

#### NCG peptides regulate physiological activities

A group of polypeptides derived from NCGs is reported to finely adjust the normal activities of muscle. The transcript of the peptide DWORF (Table 1) is annotated as a lncRNA in both mice and humans. DWORF is mainly distributed in the heart and interacts with SERCA, similarly to the SLN, PLN, MLN, and SCL peptides. It should be noted that the MLN peptide is expressed in all skeletal muscles,^72^ and the SCL peptide is expressed in somatic muscles and the postembryonic heart.^73^ DWORF can alleviate the inhibitory effects of these four peptides on SERCA in vitro. In vivo, DWORF, and PLN together maintain the dynamic regulation of cardiomyocyte contractility by competing with each other, thereby enhancing the heart pumping function during changes in the external environment.^74^ This function exemplifies a typical case of the finely tuned regulation by small molecules, namely, NGC peptides. NGC peptides are also important at the level of cell biology. LINC01420/LOC550643 RNA is thought of a noncoding RNA, but in fact, it encodes a nonannotated polypeptide referred to as P-body dissociating polypeptide (NoBody) (Table 1). This peptide is negatively correlated with the number of P-bodies. In addition, NoBody can directly contact the enhancer of decapping 4 protein (EDC4) to induce the degradation of the substrate during nonsense-mediated decay (NMD).^75^ NCG peptides can also affect cellular metabolism. As described above, MOTS-c has a significant impact on the expression of metabolism- and inflammation-associated genes. MOTS-c treatment prevents diet-induced obesity and age- or high-fat diet-associated insulin resistance in mice. MPM/Mtln extensively fine-tunes the mitochondrial membrane potential, Ca^2+^ metabolism capacity, and ROS levels, and it enhances the stability and assembly of functional complexes as a molecular chaperone on the mitochondrial membrane, thereby strengthening respiratory efficiency.^76^ Mtln also cooperates with Cyb5r3 to affect lipid metabolism. The weakening of complex I in the respiratory supercomplex in Mtln-knockout mice may also contribute to the changes in Cyb5r3-related lipid metabolism that are caused by a lack of Mtln.^77^ MOXI, the homologous peptide of MPM/Mtln in mice, regulates mitochondrial oxidation and energy homeostasis by enhancing fatty acid β-oxidation, thereby improving exercise tolerance.^78^ Two proteins that interact directly with Mtln have been found through IP assays (in refs. ^77,78^); however, the full scope of the phenotypic changes cannot be explained solely by changes in the expression of Mtln led by two proteins. Further exploration of the mechanism of MPM/Mtln/MOXI action is likely to reveal other action mechanisms, which further illustrates the importance of NCG-peptide studies.

#### NCG peptides participate in the stress response and promote tissue repair

**Fig. 6:**
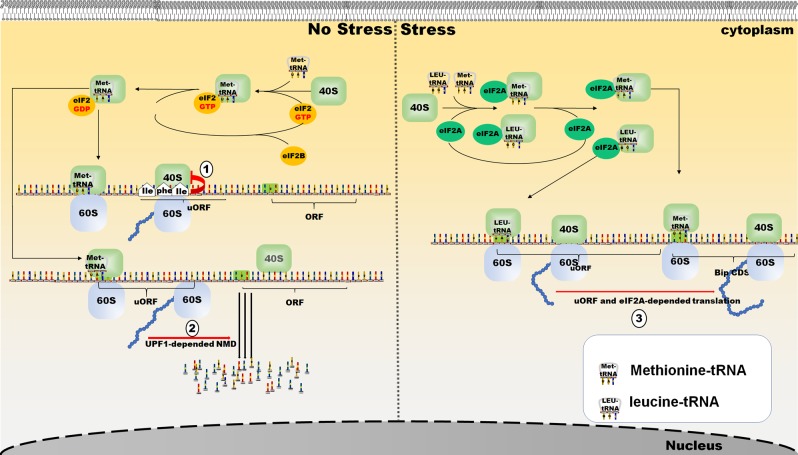
uORF can participate in the ISR reaction in three ways to facilitate the expression of genes that alleviate stress damage or trigger apoptosis. In the absence of stress, the uORF is translated to inhibit the expression of a coding-region protein by means of ribosome stalling (1) and promoting UPF1-dependent mRNA decay (2). Upon stress, uORF expression is downregulated, and inhibition is reduced, resulting in increased protein expression in the coding region. In addition, stress upregulates eIF2A levels,^140^ and leads to the constitutive translation of uORF, which promotes translation of the coding-region proteins (3).

When cells are exposed to obvious environmental changes or macromolecular damages, they can undergo a series of adaptive changes, which have an impact on gene expression to enhance the ability of damage resistance and viability under adverse conditions.^79,80^ A set of regulatory systems contribute to changes in gene expression,^81,82^ and now NCG peptides can be added to this set. A sequence-conserved uORF in the 5′UTR of the mRNA of C/EBP-homologous protein (CHOP) can be translated into peptide of 31 aa or 34 aa (Table 1), which inhibits the translation of the downstream ORF of the CHOP protein under stress-free conditions.^83^ However, under stress conditions, phosphorylation of eIF2 reduces the level of uORF translation, thereby relieving the inhibitory effect. Thus, the CHOP expression level is relatively increased.^84^ Although two uORFs are involved in the regulation of activating transcription factor 4 (ATF4), similar mechanisms are also involved. The ribosome scanning from the 5′UTR of the mRNA first encounters uORF1 and then uORF2. The two uORFs are far from each other, therefore, both can be translated. However, due to the close proximity of uORF2 to the main downstream ATF4 ORF, the ribosome cannot restore the ability to reinitiation in time, and as a consequence, the start codon of the main downstream ATF4 ORF is skipped and AFTF4 is not translated. Under stress conditions, ribosome reinitiation is even less efficient: after the translation of uORF1, the ribosomes cannot reassemble at the start codon of uORF2, and consequently, uORF2 is skipped. In contrast, some ribosomes reassemble before encountering the main ATF4 ORF, resulting in ATF4 expression.^85^ To analyze the effect of uORFs, the starting site and distance to the main ORF should be taken into consideration. In addition, inhibition of uORF translation abolishes the UPF1-dependent nonsense-mediated mRNA decay (NMD), improving the stability of IFRD1 mRNA under stress conditions.^86^ In addition, an uORF in the 5′UTR of the mRNA of binding immunoglobulin protein (BiP) can be translated into a peptide of 9 aa (Table 1) in a leucine-initiated and eIF2A-dependent nontraditional manner of translation during the stress response, promoting Bip translation during stress.^29^ In fact, many translation initiation sites of uORFs in the 5′UTR are noncanonical and may represent other action mechanisms of uORFs in an integrated stress response (ISR) (Fig. 6).^87,88^

NCG-derived peptides participate in stress in a variety of ways to protect against external damage. Once damage occurs, other NCG peptides can promote tissue repair through different mechanisms. SPAR, which is translated from LINC00961, stabilizes the v-ATPase–Ragulator–Rags supercomplex to suppress mTORC1 activation in response to amino acid stimulation. When the muscle is damaged by the external environmental stimuli, the expression of SPAR peptide (Table 1) is suppressed, upregulating the mTORC1 signaling pathway, which promotes damage repair and tissue regeneration.^89^ The aforementioned Minion/Myomixer protein is undetectable in an adult mouse without injury but becomes significantly upregulated during tissue regeneration. Mechanically, Minion/Myomixer and Myomaker together induce cell fusion to promote muscle regeneration.^65,66^

#### NCG peptides modulate tumor development

**Fig. 7:**
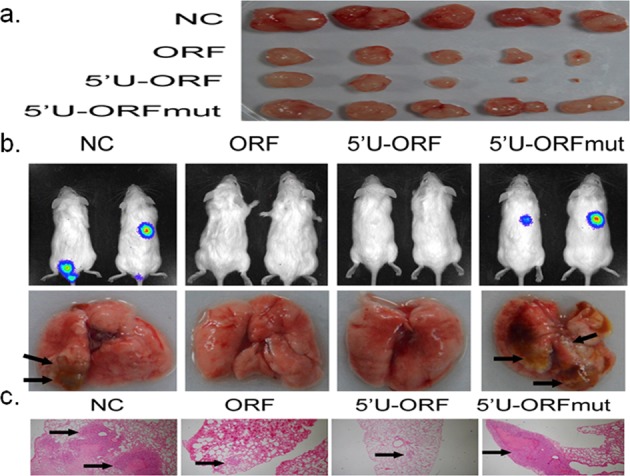
HOXB-AS3 peptides, instead of HOXB-AS3 lncRNA, suppress tumor development. The HOXB-AS3 ORF, 5′UTR-ORF, and 5′UTR-ORFmut constructs were generated to study the effect of the HOXB-AS3 peptide and lncRNA on cancer progression. Both the HOXB-AS3 ORF and 5′UTR-ORF constructs expressed the HOXB-AS3 peptide. The 5′UTR-ORFmut contains the mutated HOXB-AS3 start codon and therefore did not encode the HOXB-AS3 peptide. All of these constructs were transfected into CRC cells, which rarely express HOXB-AS3 peptides. The NC group is a negative control, which was not transfected by constructs. **a** Tumor cell xenograft assay showing that the in vivo growth of tumor cells in the NC and 5′UTR-ORFmut groups was better than it was in the ORF and 5′UTR-ORF groups. **b** Metastatic tumor model by tail vein injection showing that tumor metastasis is also promoted in the HOXB-AS3 ORF and 5′UTR-ORF group. **c** Histological analysis of the pulmonary metastasis lesion shown in **b**.

Thus far, the mechanism of tumorigenesis has not been fully elucidated. However, an increasing number of mechanisms have been explored,^90,91^ including those involved in the role of NGC peptides. Reversion of pyruvate kinase M1 (PKM1) to PKM2 is common in cancers that benefits aerobic glycolysis and creates an advantage for tumorigenesis.^92,93^ HnRNP A1 is a kind of splicing factor that inhibits the inclusion of exon 9 in pyruvate kinase M, which promotes the formation of PKM2.^94,95^ LncRNA HOXB-AS3 can be translated into a peptide of 53 aa (Table 1) that can bind directly to the RGG domain in hnRNP A1, promoting hnRNP A1 to bind to exon 9 of PKM mRNA and thus inhibit the formation of PKM2 to induce a tumor-suppression effect.^96^ Thus, HOXB-AS3 peptides, in lieu of lncRNA HOXB-AS3, play a competitive role to inhibit tumor formation, providing another example of NCG-peptide function through direct binding to another protein (Fig. 7). In addition, circPPP1R12A promotes the proliferation, migration, and invasion of cancer cells to enhance tumorigenesis and the metastasis of colon cancer by activating the Hippo-YAP signaling pathway.^97^ In addition, SHPRH-146aa and FBXW7-185aa both act as tumor-suppressor genes and can be used as independent prognostic markers.^38,39^ β-catenin-370aa acts as an oncogene to contribute to the activation of the Wnt pathway and consequently promotes liver cancer growth and metastasis by protecting full-length β-catenin from GSK3β-mediated degradation.^40^ The transcript of cancer associated with small integral membrane open-reading frame 1 (termed CASIMO1) is considered to have no coding function, but actually encodes an 84 aa integral membrane microprotein (Table 1). The CASIMO1 peptide can promote cell proliferation through the downstream SQLE/MAPK/ERK signaling pathway and induce an increase in the proportion of cells in the proliferative phase. In addition, CASIMO1 also affects the migration capacity of tumor cell lines by affecting the cytoskeleton.^98^ Pseudogenes are protein-coding genes, and loss of selection pressure causes them to undergo deleterious mutations, resulting in tissue degeneration and their eventual transition into genetic fossils.^99,100^ However, among the 11 pseudogenes of Nanog, NANOGP8 is expressed in multiple cancer cell lines and tissues,^101^ where it plays an important role in tumor development.^102^

### Pathogenicity and the potential of NCG peptides in target therapy

The pathogenesis of a large number of diseases is still unclear, and concurrently, their treatment is not satisfactory. NCG peptides may support a new perspective from which to view the underlying mechanism of diseases. Taking the above-mentioned CASIMO1 peptide, circPPP1R12A-73aa and β-catenin-370aa as examples, aberrant expression of human endogenous NCG peptides could cause diseases, including cancer. NCG peptides derived from pathogenic microorganisms can also promote the development of diseases. The E7 protein encoded by HPV virus-derived circE7 can promote the growth and tumorigenic ability of CaSki cervical carcinoma cells, while circE7 by itself cannot.^103^

In addition to providing a new perspective on pathogenicity, NCG peptides are also promising targets for targeted therapy. Some achievements have been made in this regard. MOTS-c peptide treatment can inhibit osteolysis in a mouse model, which has potential in the therapy of osteolysis and other inflammation disorders.^104^ MOTS-c peptide treatment can also increase the ability of cold adaptation upon acute cold exposure and provide a potentially therapeutic drug for cold stress-related diseases.^105^ In addition, the role of MPM in mitochondrial respiration and muscle formation makes MPM a potential target for muscular dystrophy therapy.^69^ In terms of tumor-targeted therapy, NCG peptides, such as the SHPRH-146aa, FBXW7-185aa, and HOXB-AS3 peptides, can serve as tumor-targeting therapeutic drugs. The same is true for PINT87aa. Linc-PINT simultaneously generates a circular-form circPINTexon2, and circPINTexon2 produces an 87-amino acid peptide, PINT87aa. PINT87aa directly binds to polymerase-associated factor complex (PAF1c) and inhibits several oncogenes downstream of PAF1c, including CEBP1, cyclin D1, C-myc, Sox2, etc. In biological function, PINT87aa overexpression can suppress glioblastoma in vitro and in vivo.^106^ An ideal targeted therapeutic drug should effectively kill or inhibit tumor cells while not damaging normal tissue cells. These antitumor NCG peptides are naturally targeted therapeutic drugs with significantly reduced cytotoxicity, compared with the cytotoxicity induced by traditional drugs, as and substantially reduced immunogenicity. Furthermore, a relatively smaller molecular weight makes them more likely than traditional tumor suppressive proteins to be developed into drugs. With the development of applicable materials, these peptides can be packaged by suitable carriers and delivered into tumor cells, where they can specifically inhibit tumor cells.^107^ NCG peptides also have great potential in tumor immunotherapy. The ideal tumor-specific antigens (TSAs) enable T lymphocytes to correctly recognize tumor cells, and the ideal tumor-specific antigen is a key factor in the field of immunotherapy. In a genome-wide search for TSAs, NCG peptides were found to be main sources of targetable TSAs. Tumor vaccines developed according to NCG peptides enable mice to resist tumors, suggesting that NCG peptides can be used as therapeutic targets in tumor immunotherapy, particularly in tumor vaccines.^108,109^

### Challenges and future trends

#### NCG peptides challenge the known features of coding genes

The originally discovered NCGs were found to act in the form of noncoding transcripts rather than through translation into peptides or proteins.^110–112^ However, later, some NCGs were found to have coding functions and thus should have been defined as coding genes. For example, pri-miRNAs, the primary transcripts of miRNAs and defined as NCGs, can encode peptide products.^113^ Pri-miRNAs have structures similar to traditional mRNAs, including a 5′-cap and a 3′-poly(A) tail.^114^ Taking pri-miR165a and pri-miR171b as NCG examples, they can be translated into peptides (Table 1) to promote the transcription of themselves. Further analysis shows that both are “ancient miRNAs”,^115^ which are conserved across many species, not “recent miRNAs”, which are more species-specific.^116^ Together with circRNA-derived SHPRH-146aa and FBWX7-185aa, the corresponding genes for mitochondrial genome-derived MOTS-c, lncRNA-derived MLN and DWORF, etc., were previously defined as NCGs but are capable of coding peptides. The discovery of these properties challenges previous opinions generated in NCG research and the known features of coding genes.

In addition, some NCGs have dual roles. Under some conditions, they function as a NCG, but in other conditions, they encode peptides. For example, in Drosophila melanogaster, Oscar plays its role through translation into proteins in an embryonic stage,^117,118^ and acts as a noncoding RNA during early oogenesis.^119^ In mammals, the SRA gene, which is regarded as an NCG, plays an important role in coactivating nuclear receptors^120,121^ and enhancing transcriptional factors.^122^ A new isoform, SRA1, has been found to act both as a NCG and a coding gene, and the two gene states coexist in the same cells.^123^ For this type of NCG, many questions remain unanswered. For example, under what circumstances do NCGs function as NCGs. and when are they translatable into functional peptides? What factors regulate the balance of the coding and noncoding forms? These questions are also applicable to the gene of pri, which is only expressed at a specific stage during embryonic development. For example, the Minion/Myomixer peptide is absent in uninjured muscle, but present in injured muscle.^68^ In another example, the CASIMO1 peptide is upregulated in tumors and contributes to tumorigenesis, but is downregulated in healthy tissues.^98^ Therefore, it is of paramount importance to understand the mechanisms and factors by which the NCGs switch between coding and noncoding forms and the conditions under which NCG-peptide expression is promoted or inhibited. Thus, gaining such an understanding is a great challenge and should also be a future area of focus.

#### Both the exact number and regulation mechanism remain unclear

The traditionally defined NCGs constitute >90% of the whole genome. However, the exact number of potentially coding NCGs remains unclear. Two approaches are mainly used in the search for peptides encoded by NCGs. One is to predict the coding potential of NCGs by bioinformatics analysis followed by experimental confirmation,^124^ and another is to characterize the peptides by mass spectrometry and then relate them back to genome DNA.^125^

In the first approach, bioinformatics analysis helps to target-specific genes for further confirmation and is the basis for consequent experiments. However, many puzzles confound the success of this approach. For instance, what are the characteristics of NCGs that can encode peptides? When the transcripts bind to a ribosome, is it translated into a functional peptide or is translation randomly undertaken because of probabilistic binding? In addition, a unanimous standard is demanded to facilitate the research by this approach. In the second approach, because the NCG-peptide products are more tissue-specific or state-specific than are traditional functional proteins, NCG peptides are more easily affected by extracellular stimuli. Thus, exploring the expression of NCGs only in an unstressed state or in specific cell lines may result in many peptides being undiscovered. For example, the translation of linc00689-derived micropeptide, STORM (stress- and TNF-α-activated ORF micropeptide) (Table 1), depends on eIF4E phosphorylation after TNF-α activates mammalian Ste20-like kinase (MST1).^126^ The discovery of this peptide is missed if only mass spectrometry is used to map the protein profiles in a resting state. At the same time, with an in-depth study of the coding mechanism, it is very likely to discover new mechanisms and new models of peptide translation, thus perfecting and enriching the central law, such as the non-AUG-initiated translation mechanism.^127,128^ Furthermore, the non-AUG-initiated translation mechanism of repeat polypeptides in some NCGs can directly cause diseases.^129,130^ In addition, the loop structure of circRNAs enables them to reverse the sequence of the start codon and stop codon in the gene sequence, which greatly enrich the number of ORFs.^34^

Therefore, the development of bioinformatic analysis standards and the establishment of experimental verification systems will also be a future challenge in this field. We need to explore the peptides in a boarder context to identify and characterize them.

#### Hidden functions and applications need to be uncovered

Gene expression is regulated at multiple levels. Compared with the regulation of mRNA levels, the regulation of protein levels does not involve changes in protein quantity. NCG peptides interact directly with functional proteins and thus adapt to short-term extracellular effects, and the regulation of the mRNA level is more biased to long-term adaption. Therefore, the regulation of NCG peptides in gene expression needs to be further explored. NCG peptides vary in length and are flexible in functional mechanisms. The mRNA corresponds to functional proteins. It remains unknown whether we can group peptides with the same action modes, such as MLN, DWORF, and Nobody. These NCG peptides function by affecting structural proteins, and thus, we believe that they can be named nonstructural functional peptides. Moreover, whether this mode of action is a universal mechanism for NCG peptides is currently unknown. Hence, research on the action modes and mechanisms of these peptides will also be a challenge in the future. There are significantly more NCGs than coding genes.^131^ With the continuous exploration of new mechanisms and new models, an increasing number of peptides will be discovered. The number of such peptides is possibly much larger than that of the proteins or peptide molecules we have discovered thus far. On the one hand, NCG peptides provide a new key to the door to open the mystery of life. On the other hand, they may become therapeutic targets for disease treatment. Because of their time- or tissue-specificity, NCG-encoded peptides are also time-specific and expressed in specific disease states. Hence, NCG peptides provide potential targets for disease interventions. However, these efforts have not yet begun. With the in-depth study of NCG peptides, our understanding, in either organism development or disease intervention, including tumor treatment, will surely enter a new era.

### Potential applications of NCG peptides in real-world studies

A real-word study (RWS) supplements the data obtained from traditional clinical trials.^132,133^ NCG-peptide research is still in its infancy, and medical products of NCG peptides have not yet been used in RWS research. More efforts should be made to achieve clinical translation of NCG peptides. Since nonintervention is a feature of RWS, experimental intervention is indispensable in the search for NCG peptides. How to explore the role of NCG peptides in the natural state will continue to be a challenge.

## Concluding remarks

An increasing number of NCGs have been verified to have coding functions,^134,135^ providing an in-depth understanding of life activities and complementing the existing library of protein or peptide molecules. Epigenetics and alternative splicing have indicated that the complicated human genome is even more intricate than originally thought.^136,137^ The emergence of noncoding RNA opens up a new world for the regulation of protein expression, greatly enriching the complexity of life activities.^138,139^ NCGs can also encode peptides, which undoubtedly adds a new direction for a more in-depth interpretation of the inherent laws of life. As more NCG peptides are discovered, new mechanisms and key molecules are likely to be accordingly revealed. The success in this effort with help us not only to explain the regulation process of many physiological and pathological phenomena but also to bring new ideas that promote the understanding and intervention of diseases.

## References

[1] Crick F (1970). Central dogma of molecular biology. Nature.

[2] Li JJ, Biggin MD (2015). Gene expression. Statistics requantitates the central dogma. Science.

[3] Hangauer M, Vaughn I, McManus M (2013). Pervasive transcription of the human genome produces thousands of previously unidentified long intergenic noncoding RNAs. PLoS Genet..

[4] Doolittle WF (2013). Is junk DNA bunk? A critique of ENCODE. Proc. Natl Acad. Sci. USA.

[5] Chen R (2017). Quantitative proteomics reveals that long non-coding RNA MALAT1 interacts with DBC1 to regulate p53 acetylation. Nucleic Acids Res.

[6] Y L (2016). HBXIP and LSD1 scaffolded by lncRNA hotair mediate transcriptional activation by c-Myc. Cancer Res..

[7] Hansen TB (2013). Natural RNA circles function as efficient microRNA sponges. Nature.

[8] Helwak, A., Kudla, G., Dudnakova, T. & Tollervey, D. Mapping the human miRNA interactome by CLASH reveals frequent noncanonical binding. *Cell* **153**, 654–665 (2013). 10.1016/j.cell.2013.03.043 PMC3650559 23622248

[9] Matera AG, Terns RM, Terns MP (2007). Non-coding RNAs: lessons from the small nuclear and small nucleolar RNAs. Nat. Rev. Mol. Cell Biol..

[10] Rupaimoole R, Slack FJ (2017). MicroRNA therapeutics: towards a new era for the management of cancer and other diseases. Nat. Rev. Drug Discov..

[11] Lu TX, Rothenberg ME (2018). MicroRNA. J. Allergy Clin. Immunol..

[12] Fischer JW, Leung AK (2017). CircRNAs: a regulator of cellular stress. Crit. Rev. Biochem. Mol. Biol..

[13] Salzman J (2016). Circular RNA expression: its potential regulation and function. Trends Genet..

[14] Quinn JJ, Chang HY (2016). Unique features of long non-coding RNA biogenesis and function. Nat. Rev. Genet..

[15] Mattick JS (2001). Non-coding RNAs: the architects of eukaryotic complexity. EMBO Rep..

[16] Cech T, Steitz J (2014). The noncoding RNA revolution-trashing old rules to forge new ones. Cell.

[17] Ingolia NT (2016). Ribosome footprint profiling of translation throughout the genome. Cell.

[18] Kim MS (2014). A draft map of the human proteome. Nature.

[19] Legnini I (2017). Circ-ZNF609 is a circular RNA that can be translated and functions in myogenesis. Mol. Cell.

[20] Slavoff SA (2013). Peptidomic discovery of short open reading frame-encoded peptides in human cells. Nat. Chem. Biol..

[21] Pang Y, Mao C, Liu S (2018). Encoding activities of non-coding RNAs. Theranostics.

[22] Galindo MI (2007). Peptides encoded by short ORFs control development and define a new eukaryotic gene family. PLoS Biol..

[23] Marsh JA, Teichmann SA (2015). Structure, dynamics, assembly, and evolution of protein complexes. Annu. Rev. Biochem..

[24] Kister AE, Finkelstein AV, Gelfand IM (2002). Common features in structures and sequences of sandwich-like proteins. Proc. Natl Acad. Sci. USA.

[25] Pauling L, Corey RB, Branson HR (1951). The structure of proteins; two hydrogen-bonded helical configurations of the polypeptide chain. Proc. Natl Acad. Sci. USA.

[26] Ghoorah AW, Devignes MD, Smail-Tabbone M, Ritchie DW (2014). KBDOCK 2013: a spatial classification of 3D protein domain family interactions. Nucleic Acids Res..

[27] Pugalenthi G, Bhaduri A, Sowdhamini R (2005). GenDiS: genomic distribution of protein structural domain superfamilies. Nucleic Acids Res..

[28] Levy Emmanuel D. (2007). PiQSi: Protein Quaternary Structure Investigation. Structure.

[29] Starck S (2016). Translation from the 5' untranslated region shapes the integrated stress response. Science.

[30] Couso JP, Patraquim P (2017). Classification and function of small open reading frames. Nat. Rev. Mol. Cell Biol..

[31] Hershey, J. W., Sonenberg, N. & Mathews, M. B. Principles of translational control: an overview. *Cold Spring Harb. Perspect. Biol*. **4**, a011528 (2012). 10.1101/cshperspect.a011528 PMC3504442 23209153

[32] Archer SK, Shirokikh NE, Beilharz TH, Preiss T (2016). Dynamics of ribosome scanning and recycling revealed by translation complex profiling. Nature.

[33] Hayashi N (2017). Identification of Arabidopsis thaliana upstream open reading frames encoding peptide sequences that cause ribosomal arrest. Nucleic Acids Res.

[34] Conn SJ (2015). The RNA binding protein quaking regulates formation of circRNAs. Cell.

[35] Zhou B, Yu JW (2017). A novel identified circular RNA, circRNA_010567, promotes myocardial fibrosis via suppressing miR-141 by targeting TGF-beta1. Biochemical Biophysical Res. Commun..

[36] Unk I (2006). Human SHPRH is a ubiquitin ligase for Mms2-Ubc13-dependent polyubiquitylation of proliferating cell nuclear antigen. Proc. Natl Acad. Sci. USA.

[37] Motegi A (2008). Polyubiquitination of proliferating cell nuclear antigen by HLTF and SHPRH prevents genomic instability from stalled replication forks. Proc. Natl Acad. Sci. USA.

[38] Zhang M (2018). A novel protein encoded by the circular form of the SHPRH gene suppresses glioma tumorigenesis. Oncogene.

[39] Yang Yibing, Gao Xinya, Zhang Maolei, Yan Sheng, Sun Chengjun, Xiao Feizhe, Huang Nunu, Yang Xuesong, Zhao Kun, Zhou Huangkai, Huang Suyun, Xie Bo, Zhang Nu (2017). Novel Role of FBXW7 Circular RNA in Repressing Glioma Tumorigenesis. JNCI: Journal of the National Cancer Institute.

[40] Liang WC (2019). Translation of the circular RNA circbeta-catenin promotes liver cancer cell growth through activation of the Wnt pathway. Genome Biol..

[41] Anderson D (2015). A micropeptide encoded by a putative long noncoding RNA regulates muscle performance. Cell.

[42] Wei J, Wu C, Sachs MS (2012). The arginine attenuator peptide interferes with the ribosome peptidyl transferase center. Mol. Cell. Biol.

[43] Bhushan S (2010). Structural basis for translational stalling by human cytomegalovirus and fungal arginine attenuator peptide. Mol. Cell.

[44] Hinnebusch A, Ivanov I, Sonenberg N (2016). Translational control by 5'-untranslated regions of eukaryotic mRNAs. Science.

[45] Inagaki S (2005). Identification and expression analysis of putative mRNA-like non-coding RNA in Drosophila. Genes Cells : Devoted Mol. Cell. Mechanisms.

[46] Kondo T (2007). Small peptide regulators of actin-based cell morphogenesis encoded by a polycistronic mRNA. Nat. Cell Biol..

[47] Zanet J (2015). Pri sORF peptides induce selective proteasome-mediated protein processing. Science.

[48] Breton S (2014). A resourceful genome: updating the functional repertoire and evolutionary role of animal mitochondrial DNAs. Trends Genet..

[49] Gissi C, Iannelli F, Pesole G (2008). Evolution of the mitochondrial genome of Metazoa as exemplified by comparison of congeneric species. Heredity.

[50] Mercer TR (2011). The human mitochondrial transcriptome. Cell.

[51] Lee C (2015). The mitochondrial-derived peptide MOTS-c promotes metabolic homeostasis and reduces obesity and insulin resistance. Cell Metab..

[52] Pauli A (2014). Toddler: an embryonic signal that promotes cell movement via Apelin receptors. Science.

[53] Yang Y (2017). Extensive translation of circular RNAs driven by N-methyladenosine. Cell Res..

[54] Zhou J (2015). Dynamic m(6)A mRNA methylation directs translational control of heat shock response. Nature.

[55] Coots RA (2017). m(6)A Facilitates eIF4F-Independent mRNA Translation. Mol. Cell.

[56] Wang Y, Wang Z (2015). Efficient backsplicing produces translatable circular mRNAs. RNA.

[57] Kirchberber MA, Tada M, Katz AM (1975). Phospholamban: a regulatory protein of the cardiac sarcoplasmic reticulum. Recent Adv. Stud. Card. Struct. Metab..

[58] Wawrzynow A (1992). Sarcolipin, the “proteolipid” of skeletal muscle sarcoplasmic reticulum, is a unique, amphipathic, 31-residue peptide. Arch. Biochem. Biophysics..

[59] Kranias E, Hajjar R (2012). Modulation of cardiac contractility by the phospholamban/SERCA2a regulatome. Circ. Res..

[60] Schmitt JP (2003). Dilated cardiomyopathy and heart failure caused by a mutation in phospholamban. Science.

[61] Haghighi K (2012). The human phospholamban Arg14-deletion mutant localizes to plasma membrane and interacts with the Na/K-ATPase. J. Mol. Cell. Cardiol..

[62] Velasco S (2017). A multi-step transcriptional and chromatin state cascade underlies motor neuron programming from embryonic stem cells. Cell Stem Cell.

[63] Flynn RA, Chang HY (2014). Long noncoding RNAs in cell-fate programming and reprogramming. Cell Stem Cell.

[64] Kondo T (2010). Small peptides switch the transcriptional activity of Shavenbaby during Drosophila embryogenesis. Science.

[65] Zhang Q (2017). The microprotein Minion controls cell fusion and muscle formation. Nat. Commun..

[66] Bi P (2017). Control of muscle formation by the fusogenic micropeptide myomixer. Science.

[67] Shi J (2017). Requirement of the fusogenic micropeptide myomixer for muscle formation in zebrafish. Proc. Natl Acad. Sci. USA.

[68] Bi P (2018). Fusogenic micropeptide Myomixer is essential for satellite cell fusion and muscle regeneration. Proc. Natl Acad. Sci. USA.

[69] Lin YF (2019). A novel mitochondrial micropeptide MPM enhances mitochondrial respiratory activity and promotes myogenic differentiation.. Cell Death Dis..

[70] Norris, M. et al. Toddler signaling regulates mesodermal cell migration downstream of Nodal signaling. *eLife* **6**, e22626 (2017). 10.7554/eLife.22626 PMC5679751 29117894

[71] Freyer L (2017). Loss of apela peptide in mice causes low penetrance embryonic lethality and defects in early mesodermal derivatives. Cell Rep.

[72] DM A (2016). Widespread control of calcium signaling by a family of SERCA-inhibiting micropeptides. Sci. Signal..

[73] Magny E (2013). Conserved regulation of cardiac calcium uptake by peptides encoded in small open reading frames. Science.

[74] Nelson BR (2016). A peptide encoded by a transcript annotated as long noncoding RNA enhances SERCA activity in muscle. Science.

[75] D’Lima N (2017). A human microprotein that interacts with the mRNA decapping complex. Nat. Chem. Biol..

[76] Stein CS (2018). Mitoregulin: a lncRNA-encoded microprotein that supports mitochondrial supercomplexes and respiratory efficiency. Cell Rep..

[77] Chugunova A, Loseva E, Mazin P (2019). LINC00116 codes for a mitochondrial peptide linking respiration and lipid metabolism. Proc. Natl Acad. Sci. USA.

[78] Makarewich CA (2018). MOXI is a mitochondrial micropeptide that enhances fatty acid beta-oxidation. Cell Rep.

[79] Akerfelt M, Morimoto RI, Sistonen L (2010). Heat shock factors: integrators of cell stress, development and lifespan. Nat. Rev. Mol. cell Biol..

[80] Thomas MP, Lieberman J (2013). Live or let die: posttranscriptional gene regulation in cell stress and cell death. Immunological Rev..

[81] Kaplan KB, Li R (2012). A prescription for ‘stress’-the role of Hsp90 in genome stability and cellular adaptation. Trends Cell Biol..

[82] Detzer A, Engel C, Wunsche W, Sczakiel G (2011). Cell stress is related to re-localization of Argonaute 2 and to decreased RNA interference in human cells. Nucleic Acids Res.

[83] Jousse C (2001). Inhibition of CHOP translation by a peptide encoded by an open reading frame localized in the chop 5'UTR. Nucleic Acids Res.

[84] Palam LR, Baird TD, Wek RC (2011). Phosphorylation of eIF2 facilitates ribosomal bypass of an inhibitory upstream ORF to enhance CHOP translation. J. Biol. Chem.

[85] Vattem KM, Wek RC (2004). Reinitiation involving upstream ORFs regulates ATF4 mRNA translation in mammalian cells. Proc. Natl Acad. Sci. USA.

[86] Zhao C (2010). Stress-sensitive regulation of IFRD1 mRNA decay is mediated by an upstream open reading frame. J. Biol. Chem.

[87] Lee S (2012). Global mapping of translation initiation sites in mammalian cells at single-nucleotide resolution. Proc. Natl Acad. Sci. USA.

[88] Na CH (2018). Discovery of noncanonical translation initiation sites through mass spectrometric analysis of protein N termini. Genome Res..

[89] Matsumoto A (2017). mTORC1 and muscle regeneration are regulated by the LINC00961-encoded SPAR polypeptide. Nature.

[90] Chaffer CL, Weinberg RA (2015). How does multistep tumorigenesis really proceed?. Cancer Discov..

[91] Tomasetti C, Li L (2017). Stem cell divisions, somatic mutations, cancer etiology, and cancer prevention.. Science.

[92] Chen M, Zhang J, Manley JL (2010). Turning on a fuel switch of cancer: hnRNP proteins regulate alternative splicing of pyruvate kinase mRNA. Cancer Res.

[93] Liang J (2016). PKM2 dephosphorylation by Cdc25A promotes the Warburg effect and tumorigenesis. Nat. Commun..

[94] Chen M, David CJ, Manley JL (2012). Concentration-dependent control of pyruvate kinase M mutually exclusive splicing by hnRNP proteins. Nat. Struct. Mol. Biol..

[95] Feng J (2016). The involvement of splicing factor hnRNP A1 in UVB-induced alternative splicing of hdm2.. Photochem. Photobiol..

[96] Huang J (2017). A peptide encoded by a putative lncRNA HOXB-AS3 suppresses colon cancer growth. Mol. Cell.

[97] Zheng X (2019). A novel protein encoded by a circular RNA circPPP1R12A promotes tumor pathogenesis and metastasis of colon cancer via Hippo-YAP signaling. Mol. Cancer.

[98] Polycarpou-Schwarz Maria, Groß Matthias, Mestdagh Pieter, Schott Johanna, Grund Stefanie E., Hildenbrand Catherina, Rom Joachim, Aulmann Sebastian, Sinn Hans-Peter, Vandesompele Jo, Diederichs Sven (2018). The cancer-associated microprotein CASIMO1 controls cell proliferation and interacts with squalene epoxidase modulating lipid droplet formation. Oncogene.

[99] Kalyana-Sundaram S (2012). Expressed pseudogenes in the transcriptional landscape of human cancers. Cell.

[100] Rapicavoli NA (2013). A mammalian pseudogene lncRNA at the interface of inflammation and anti-inflammatory therapeutics. eLife.

[101] Zhang J (2006). NANOGP8 is a retrogene expressed in cancers. FEBS J..

[102] Jeter CR (2009). Functional evidence that the self-renewal gene NANOG regulates human tumor development. Stem Cells.

[103] Zhao J, Lee EE, Kim J (2019). Transforming activity of an oncoprotein-encoding circular RNA from human papillomavirus.. Nat. Commun..

[104] Yan Z (2019). MOTS-c inhibits osteolysis in the mouse Calvaria by affecting osteocyte-osteoclast crosstalk and inhibiting inflammation. Pharmacol. Res..

[105] Lu Huanyu, Tang Shan, Xue Chong, Liu Ying, Wang Jiye, Zhang Wenbin, Luo Wenjing, Chen Jingyuan (2019). Mitochondrial-Derived Peptide MOTS-c Increases Adipose Thermogenic Activation to Promote Cold Adaptation. International Journal of Molecular Sciences.

[106] Zhang M (2018). A peptide encoded by circular form of LINC-PINT suppresses oncogenic transcriptional elongation in glioblastoma.. Nat. Commun..

[107] B L (2019). RBC membrane camouflaged prussian blue nanoparticles for gamabutolin loading and combined chemo/photothermal therapy of breast cancer. Biomaterials.

[108] Laumont, C. M. & Vincent, K. Noncoding regions are the main source of targetable tumor-specific antigens. **10**, eaau5516 (2018). 10.1126/scitranslmed.aau5516 30518613

[109] Laumont CM, Perreault C (2018). Exploiting non-canonical translation to identify new targets for T cell-based cancer immunotherapy.. Cell. Mol. Life Sci..

[110] Hang J, Wan R, Yan C, Shi Y (2015). Structural basis of pre-mRNA splicing. Science.

[111] Kaida D (2010). U1 snRNP protects pre-mRNAs from premature cleavage and polyadenylation. Nature.

[112] Chen CK (2016). Xist recruits the X chromosome to the nuclear lamina to enable chromosome-wide silencing. Science.

[113] Lauressergues D (2015). Primary transcripts of microRNAs encode regulatory peptides. Nature.

[114] Lee Y (2004). MicroRNA genes are transcribed by RNA polymerase II. EMBO J.

[115] Waterhouse PM, Hellens RP (2015). Plant biology: coding in non-coding RNAs. Nature.

[116] Li X (2017). Conservation and diversification of the miR166 family in soybean and potential roles of newly identified miR166s. BMC Plant Biol..

[117] Breitwieser W, Markussen FH, Horstmann H, Ephrussi A (1996). Oskar protein interaction with Vasa represents an essential step in polar granule assembly. Genes Dev.

[118] Braat AK (2004). Localization-dependent oskar protein accumulation; control after the initiation of translation. Developmental Cell.

[119] Kanke M (2015). oskar RNA plays multiple noncoding roles to support oogenesis and maintain integrity of the germline/soma distinction. RNA.

[120] Lanz RB (1999). A steroid receptor coactivator, SRA, functions as an RNA and is present in an SRC-1 complex. Cell.

[121] Colley SM, Leedman PJ (2009). SRA and its binding partners: an expanding role for RNA-binding coregulators in nuclear receptor-mediated gene regulation. Crit. Rev. Biochem. Mol. Biol..

[122] Caretti G (2006). The RNA helicases p68/p72 and the noncoding RNA SRA are coregulators of MyoD and skeletal muscle differentiation. Developmental Cell.

[123] Hube F (2011). Steroid receptor RNA activator protein binds to and counteracts SRA RNA-mediated activation of MyoD and muscle differentiation. Nucleic Acids Res..

[124] Ingolia NT (2014). Ribosome profiling reveals pervasive translation outside of annotated protein-coding genes. Cell Rep..

[125] Wilhelm M (2014). Mass-spectrometry-based draft of the human proteome. Nature.

[126] Min KW (2017). eIF4E phosphorylation by MST1 reduces translation of a subset of mRNAs, but increases lncRNA translation. Biochimica et. Biophysica Acta Gene Regulatory Mechanisms..

[127] Starck SR (2012). Leucine-tRNA initiates at CUG start codons for protein synthesis and presentation by MHC class I. Science.

[128] Ivanov IP (2011). Identification of evolutionarily conserved non-AUG-initiated N-terminal extensions in human coding sequences. Nucleic Acids Res.

[129] Todd P (2013). CGG repeat-associated translation mediates neurodegeneration in fragile X tremor ataxia syndrome. Neuron.

[130] Mori K., Weng S.-M., Arzberger T., May S., Rentzsch K., Kremmer E., Schmid B., Kretzschmar H. A., Cruts M., Van Broeckhoven C., Haass C., Edbauer D. (2013). The C9orf72 GGGGCC Repeat Is Translated into Aggregating Dipeptide-Repeat Proteins in FTLD/ALS. Science.

[131] Elkon R, Agami R (2017). Characterization of noncoding regulatory DNA in the human genome. Nat. Biotechnol..

[132] Khozin, S., Blumenthal, G. M. & Pazdur, R. Real-world data for clinical evidence generation in oncology. *J. Natl Cancer Inst*. **109**, djx187 (2017). 10.1093/jnci/djx187 29059439

[133] Sherman RE (2016). Real-world evidence - what is it and what can it tell us?. N. Engl. J. Med.

[134] Pamudurti N (2017). Translation of CircRNAs. Mol. Cell..

[135] Andreev D (2015). Translation of 5' leaders is pervasive in genes resistant to eIF2 repression. eLife.

[136] Cheung WA (2017). Functional variation in allelic methylomes underscores a strong genetic contribution and reveals novel epigenetic alterations in the human epigenome. Genome Biol..

[137] Baralle FE, Giudice J (2017). Alternative splicing as a regulator of development and tissue identity. Nat. Rev. Mol. Cell Biol..

[138] Marchese FP, Raimondi I, Huarte M (2017). The multidimensional mechanisms of long noncoding RNA function. Genome Biol..

[139] Chen JA, Conn S (2017). Canonical mRNA is the exception, rather than the rule.. Genome Biol..

[140] Kearse MG, Wilusz JE (2017). Non-AUG translation: a new start for protein synthesis in eukaryotes. Genes Dev..

